# Supplementary material for: A realistic phantom dataset for benchmarking cryo-ET data annotation
Source: Nat Methods. 2025 Aug 26;22(9):1819–23. doi: 10.1038/s41592-025-02800-5 (PMC12446061; doi:10.1038/s41592-025-02800-5)
Supplement: Supplementary file 1 — Supplementary Tables 1–3. [file 41592_2025_2800_MOESM1_ESM.pdf]

nature methods

Supplementary information https://doi.org/10.1038/s41592-025-02800-5

A realistic phantom dataset for benchmarking cryo-ET data annotation

In the format provided by the authors and unedited

Software Package	Repository Link	License
Copick	https://github.com/copick/copick	MIT
ChimeraX-Copick	https://github.com/copick/chimerax-copick	MIT
Copicklive	https://github.com/copick/copick_live	MIT
CellCanvas	https://github.com/cellcanvas/cellcanvas	MIT
AreTomo3	https://github.com/czimaginginstitute/AreTomo3	BSD 3-Clause
DeepFindET	https://github.com/copick/DeepFindET	GPLv3
Slabpick[a]	https://github.com/apeck12/slabpick	MIT

[a] *slabpick* was used for both the slab-picking and minislab-curation workflows.

Supplementary Table 1 | Software Availability. Source code and open-source licenses for tools used for tomogram reconstruction, annotation generation, and annotation curation in this study.

Notebook	Description	Link
3D U-Net	A 3D U-Net built on MONAI that uses copick projects for training and prediction	https://github.com/czimaginginstitute/2024_czii_mlchallenge_notebooks/tree/main/3d_unet_monai
TomoTwin	An application of the generalist particle picking tool, TomoTwin, for inference.	https://github.com/czimaginginstitute/2024_czii_mlchallenge_notebooks/tree/main/tomotwin_picking_notebook
DeepFindET	A ResUNet-based model derived from DeepFinder[12] that uses copick projects for trianing and prediction.	https://github.com/czimaginginstitute/2024_czii_mlchallenge_notebooks/tree/main/DeepFindET

Supplementary Table 2 | Example notebooks. These example notebooks are based on three published models and were designed to reduce barriers for ML experts without cryoET domain knowledge. These notebooks provide a full workflow for training, predicting, and submitting to Kaggle, and they are meant to familiarize the participants with the copick libraries and metadata/data handling.

Tomogram Type	Reconstruction workflow	Description	Contrast
Weighted Back Projection	AreTomo3	Real space WBP	Low
CTF-Deconvolved	AreTomo3	Local CTF correction of tilt series+ real space WBP	Medium
Denoised	AreTomo3 +DenoisET	Local CTF correction of tilt series+ real space WBP+tomogram denoising	High
IsoNet-Corrected	AreTomo3 +DenoisET +IsoNet	Local CTF correction of tilt series+ real space WBP+tomogram denoising+missing wedge estimation and correction	High

Supplementary Table 3 | Different tomogram types available on the Data Portal. All of these tomograms have the same tilt series alignment; therefore, the particle coordinates are the same across all types.
